# Development of a miniaturized stimulation device for electrical stimulation of cells

**DOI:** 10.1186/s13036-015-0012-1

**Published:** 2015-09-04

**Authors:** Gordon Minru Xiong, Anh Tuan Do, Jun Kit Wang, Chee Leong Yeoh, Kiat Seng Yeo, Cleo Choong

**Affiliations:** School of Materials Science and Engineering, Nanyang Technological University, Block N4.1, Nanyang Avenue, 639798 Singapore; School of Electrical and Electronic Engineering, Nanyang Technological University, Block S2.1, 50 Nanyang Avenue, 639798 Singapore; Residues and Resource Reclamation Centre (R3C), Nanyang Environmental and Water Research Institute (NEWRI), 1 Cleantech Loop, 637141 Singapore; Interdisciplinary Graduate School, Nanyang Technological University, 50 Nanyang Avenue, 639798 Singapore

**Keywords:** Electrical stimulation, Stimulation device, Frequency division, Endothelial cells, Neuroblasts, Keratinocytes, Agar salt bridge

## Abstract

**Background:**

Directing cell behaviour using controllable, on-demand non-biochemical methods, such as electrical stimulation is an attractive area of research. While there exists much potential in exploring different modes of electrical stimulation and investigating a wider range of cellular phenomena that can arise from electrical stimulation, progress in this field has been slow. The reasons for this are that the stimulation techniques and customized setups utilized in past studies have not been standardized, and that current approaches to study such phenomena rely on low throughput platforms with restricted variability of waveform outputs.

**Results:**

Here, we first demonstrated how a variety of cellular responses can be elicited using different modes of DC and square waveform stimulation. Intracellular calcium levels were found to be elevated in the neuroblast cell line SH-SY5Y during stimulation with 5 V square waves and, stimulation with 150 mV/mm DC fields and 1.5 mA DC current resulted in polarization of protein kinase Akt in keratinocytes and elongation of endothelial cells, respectively. Next, a miniaturized stimulation device was developed with an integrated cell chamber array to output multiple discrete stimulation channels. A frequency dividing circuit implemented on the device provides a robust system to systematically study the effects of multiple output frequencies from a single input channel.

**Conclusion:**

We have shown the feasibility of directing cellular responses using various stimulation waveforms, and developed a modular stimulation device that allows for the investigation of multiple stimulation parameters, which previously had to be conducted with different discrete equipment or output channels. Such a device can potentially spur the development of other high throughput platforms for thorough investigation of electrical stimulation parameters on cellular responses.

## Background

The study and manipulation of cell behaviour are currently predominantly conducted with biologics or chemical factors such as protein growth factors, blocking antibodies or small molecular entities that bind to specific protein moieties in the cell. However, these biochemical agents may be difficult to synthesize and could be limited by quality and costs. In addition, the pharmacological effects of biochemical agents depend on multiple factors, which include their pharmacokinetics, interactions with other molecules in the body, clearance rates from the body and toxicity [1]. On the other hand, physical stimuli such as electrical currents provide a more controllable form of on-demand stimuli for the cells by altering the cell membrane potential and subsequent ion flux across the membrane [2, 3]. The physiological effects of electrical stimuli have been shown to be due to the redistribution of cell surface receptors and cytoskeleton reorganization in response to the applied electrical fields [4].

Specifically, electrical stimulation with direct current (DC) signals of 100–400 mV/mm have been shown to affect intracellular calcium dynamics, [5] epithelial cell proliferation, [6] cell migration, [7, 8] and stem cell differentiation [9]. Also, Zhao et al. demonstrated cathodal (−) electrotaxis for keratinocytes upon the application of a 150 mV/mm DC stimulation [7]. Similar electrotactic responses have also been observed for neural cells, adipose-derived stem cells and osteoblasts [10–12]. While DC signals have gained the attention of biomedical scientists and engineers in recent years, other waveforms such as alternating current (AC) or square waves have not been studied as extensively in non-excitable cell types. Although FDA-approved functional electrical stimulation devices (FES) that have been configured to deliver AC or square waveforms are already in clinical use for neuromuscular stimulation and wound healing [13–15], investigations into the effects of AC signals on physiological processes have yet to be elucidated.

In fact, emerging evidence of the potential of AC signals are just beginning to emerge; for instance in mesenchymal stem cell differentiation into osteoblasts [16]. Unlike continuous DC stimulation, square waveforms minimise the deleterious irreversible non-faradaic reactions at the tissue/electrode interface [17]. Charge-balanced biphasic stimuli are also an attractive option for electrical manipulation of cellular behaviour as they theoretically leave no residual charges at the site. Currently, the published studies on square and sinusoidal waveform stimulation use a wide range of waveforms that vary in stimulation mode (voltage or current mode), frequency and amplitude [16, 18–20]. Frequency-dependent neuronal responses such as elongation and neurite outgrowth are well-known [19]. The proliferation of MC3T3-E1 bone cells have been known to occur at electrical fields of 60 Hz [18], and at 10 kHz, sinusoidal electrical fields promoted mesenchymal stem cell differentiation [16]. In another study, rectangular pulses of 1 kHz were shown to induce cathodal neurite growth and branching [19]. As abovementioned, the deployment of different stimulation frequencies to probe physiological responses vary greatly, and only a few studies have been conducted to investigate them systematically.

**Fig. 1:**
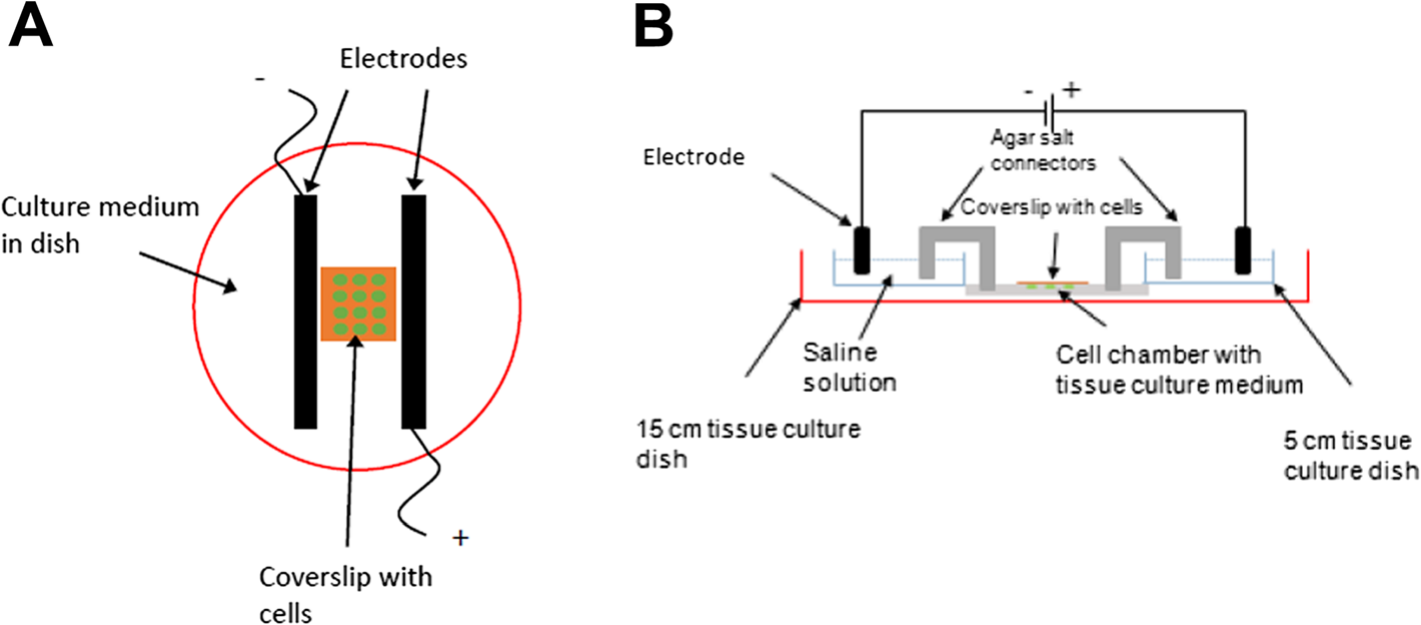
General schemes of commonly-employed experimental setups for electrical stimulation experiments. **a** Cells are seeded on coverslip placed between electrodes in tissue culture dish, and **b** Cell chamber isolated from electrodes and electrical path connected to cells via agar salt connectors

Customized setups in laboratory for electrical stimulation fall generally into 2 main categories: 1) setups constructed from petri dishes or cell chambers with a conductive element (e.g. graphite or platinum wires) directly in culture medium (Fig. 1a) [21], or 2) setups with agar/salt bridges to indirectly conduct ionic flow from electrodes to an isolated cell chamber (Fig. 1b) [22]. Biomimetic systems designed to recapitulate the extracellular environment of target cell types, such as in providing bioactive surfaces or 3-dimensional scaffolds, are also used in conjunction with electrical stimulation [17, 23]. While such setups may suffice in single-waveform stimulation investigations, they do not allow output of multiple stimulation channels in a single experiment. For example, the agar/salt bridge configuration allows for the isolation of cells undergoing stimulation from the harmful electrolytic products of the electrodes, but it occupies a large gross volume in the cell culture incubator due to its use of saline chambers and salt bridge connectors.

As such, this current study describes the use of a commercially-available stimulation board to systematically investigate physiological responses of DC and square waveforms on the intracellular localization of migration pathway-associated factor, cellular morphology and calcium signalling. Subsequently, a circuit board stimulation device integrated with a cell chamber array was designed to allow investigation into the effects of varying waveforms simultaneously on cell responses. The circuit design implements a ‘divide-by-2’ frequency divider to receive an input voltage waveform with frequency f_0_ from a waveform generator or a programmable microcontroller and give output pulses with f_0_/2, f_0_/4 and f_0_/8. Output pins next to each biocompatible chamber in the array will connect platinum wires to provide the stimuli. Using the stimulation device, the impedance frequency response of an ITO-coated glass was characterized. The miniature stimulation device is proposed to be used as a platform to study the effects of stimulation frequencies on cell behaviour and serves as a platform to develop a high throughput stimulation device with programmable output waveforms in future. In addition, a lower height profile and improved portability over existing common designs was targeted.

## Results and discussion

### Calcium signalling in SH-SY5Y cells using pulsed voltages

**Fig. 2:**
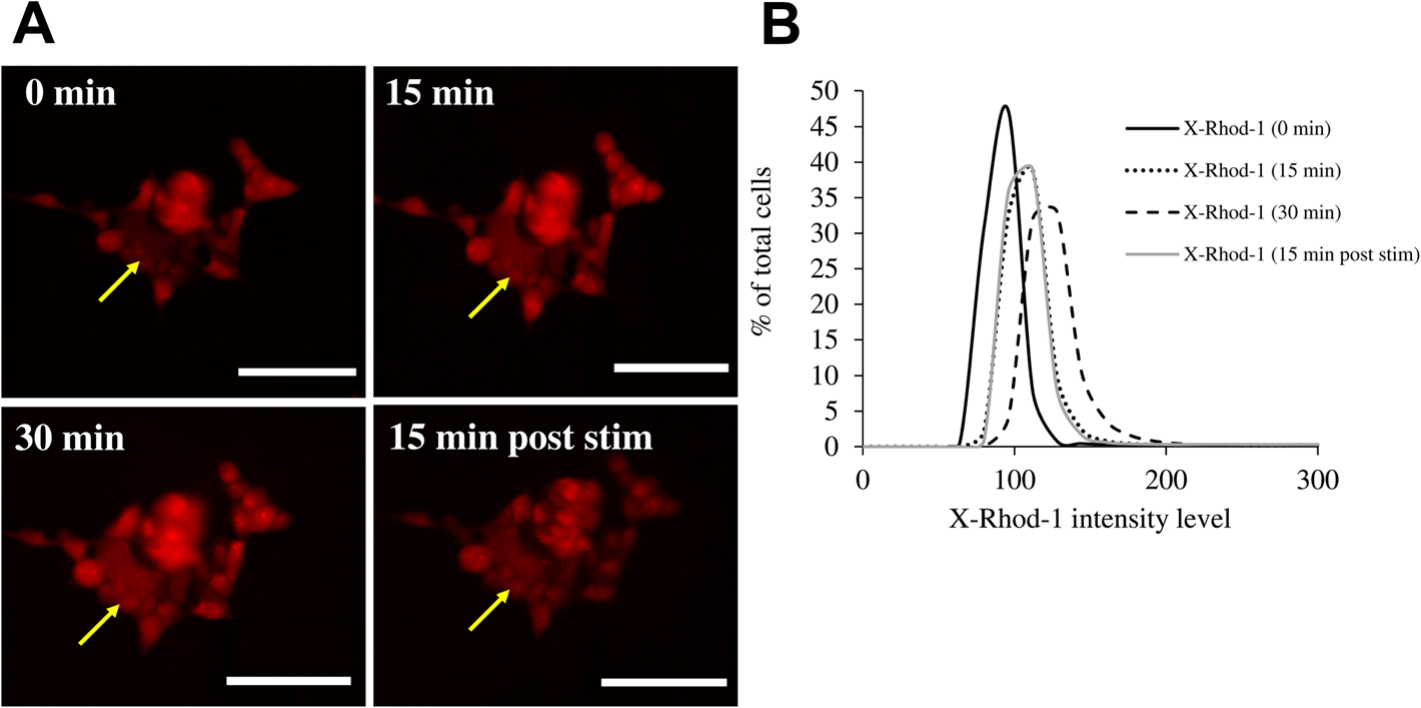
Levels of intracellular calcium in SH-SY5Y cells during stimulation with AC square waves. **a** Fluorescence images showing increase in calcium-binding dye X-Rhod-1 levels (yellow arrows) over 30 min of stimulation, and decrease in X-Rhod-1 levels 15 min after stimulation was discontinued. **b** The histograms show the corresponding intensity values of the entire field over 30 min stimulation with 5 V, 1 Hz AC square waves, and 15 min post-stimulation. Scale bar = 100 μm

The responses of neural-derived cell types towards both monophasic and biphasic pulsed stimulation have been characterized in neural stem cell differentiation [24, 25], neurite outgrowth and guidance [19]. Neuronal activity such as membrane depolarization has led to increase in intracellular calcium levels, and such calcium activity is postulated to be acting as a secondary messenger which regulates gene expression and physiological activity in neurons [26, 27]. In this study, pulsed stimulation of the neuroblastoma-derived cell line SH-SY5Y with 5 V_p-p_ monophasic, low frequency (1 Hz) and short pulses (2 msec pulse width) was conducted. The frequency of stimulation was kept low as the V_p-p_ was utilized high at 5 V; as we have used an unbalanced (monophasic) waveform, this prevents excessive build-up of charges too quickly. The calcium levels as indicated by the XRhod-1 dye intensity in the SH5Y cells increased over the 30 min of stimulation, and decreased when the stimulation was discontinued for 15 min (Fig. 2), thus demonstrating cellular response towards pulsed voltages.

### DC stimulation of PH-Akt-GFP overexpressing keratinocytes

**Fig. 3:**
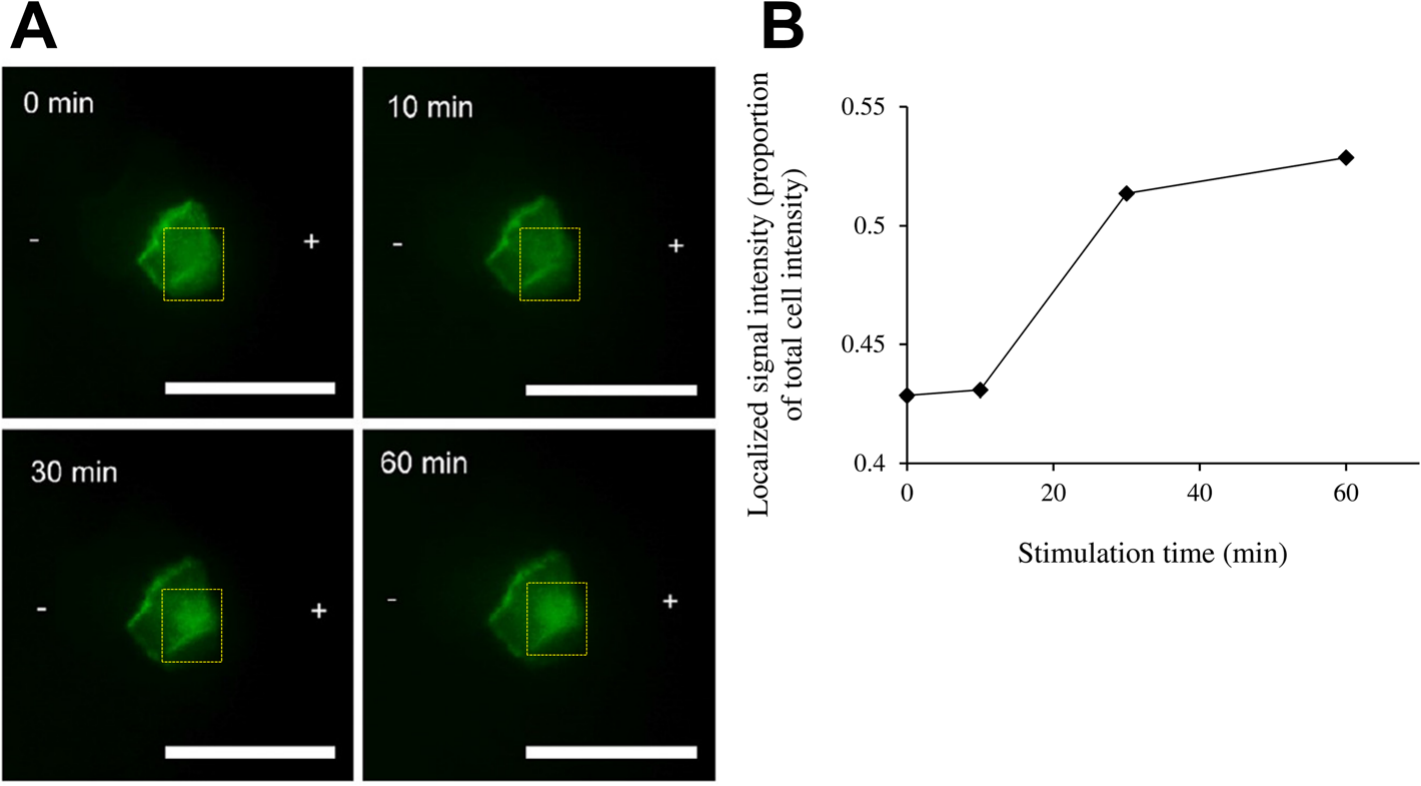
Polarization of PH-Akt-GFP in HaCaT cells under DC stimulation (150mV/mm). **a** Over time, localization of PH-Akt-GFP to the anodal side (+) of the cell was observed. **b** The localized signal intensity at the anodal end of each cell (yellow dotted box) was quantitated as a proportion of the total signal intensity of the cell. Scale bar = 50 μm

The immortalized keratinocyte cell line HaCaT was transfected with an *Akt* domain tagged with GFP. *Akt (Protein kinase B)* is a protein kinase with roles in multiple cellular processes such as proliferation and cell migration [28]. The localization of *Akt* at the edge of migrating cells has been demonstrated widely in literature, since *Akt* binds to *phosphatidylinositol (3,4,5)-trisphosphate (PIP3)* at the cell membrane, and can be phosphorylated by *phosphoinositide 3-kinase (PI3-K)* for activation [28]. The activated form of *Akt* then translocated into the cell nucleus where it controls a whole milieu of cellular processes [29]. The *PH-Akt-GFP* gene construct expresses the PH domain of the *Akt* protein, which is its *PIP3*-binding domain. In addition, GFP is tagged to *PH-Akt* for fluorescence monitoring of its localization in cell biology studies. Zhao et al. [7] has demonstrated the electrotactic response of primary keratinocytes in culture towards the cathode (−) when a DC field of 150 mV/mm was applied, and that these responses were dependent on the *Akt-PI3-K* signaling axis [7]. We applied a DC field of similar strength on HaCaT cells to study the levels and distribution of *Akt* within the cell. In contrary to cathodal migration and *Akt-PI-3 K* pathway activation at the cathodal edge observed by Zhao et al. [7] we instead observed polarization of *PH-Akt* at the anodal edge (+) of the HaCaT cells over 60 min of stimulation (Fig. 3). This difference could be due to the different migration responses exhibited by primary keratinocytes and immortalized keratinocytes [30]. It has been found that HaCaT cells, as opposed to primary keratinocytes, lack the catalytic activity of *metallopeptidase-1 (MMP-1)* and unlike the primary keratinocytes, did not migrate across the collagen type 1 substrate [30]. It is therefore feasible that differences in the direction of migration between 2 different cell types, or even between the primary and transformed line of the same cell type, could exist. Indeed, cell types that have been shown to exhibit anodal migration have been published in the literature [31, 32].

### DC stimulation of endothelial cells

**Fig. 4:**
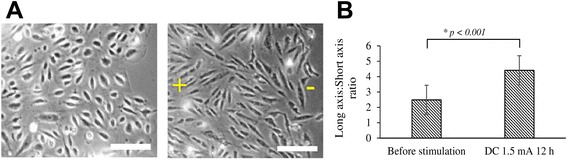
Change in endothelial cell morphology as the result of DC stimulation. **a** Elongation of HUVECs before and after 12 h stimulation with 1.5 mA DC current, and **b** Plot of the ratio of long axis : short axis , as calculated from 10 cells using ImageJ. Scale bar = 50 μm

DC stimulation of HUVECs were conducted for 12 h for the observation of any morphology change. After 12 h stimulation with a 1.5 mA current, the HUVECs were observed to have adopted an elongated morphology (Fig. 4a). The elongation of endothelial cells in response to shear stress exerted by flowing blood has been well-characterized [33, 34]. Endothelial cells have been demonstrated to undergo directional migration, reorientation and elongation under DC fields of 150–400 mV/mm [31]. In agreement with published literature, our use of current-mode DC stimulation resulted in elongation of HUVECs when quantified using long axis/short axis ratios (Fig. 4b).

### Challenges of current cell stimulation techniques

From the cell stimulation experiments, we have demonstrated the physiological responses, such as increased Ca^2+^ concentration, localisation of Akt and cell elongation, in various cell types in response to electrical stimulation. We have employed a commercially-available stimulation electrode board (C-Dish™) in combination with other discrete electrical equipment. However the C-Dish™ stimulation board was designed to be used in conjunction with the C-Pace EP Cell Stimulator (IonOptix, USA), with pulsed waveforms ranging from 1–100 Hz. Although the stimulation board fits multi-well culture plates, only a single waveform could be used for them simultaneously, as all electrodes will receive signals from a single channel on the stimulator. Current cell stimulators on the market meant for biological use are limited to DC and pulsed voltages (monophasic and biphasic), and do not have kHz frequency outputs. This is consistent with that fact that most cell stimulation experiments today only require DC or low frequency waveforms [17, 23–26]. The exploration of high frequency waveforms and their physiological effects have only been explored in setups similar to ours that utilize a discrete waveform generator [16, 25]. The disadvantage of using a waveform generator is that they are designed for use with electronic circuits with fixed resistances/impedances. Cellular studies frequently require voltage- or current-controlled outputs that can compensate for changes in biological responses as the culture medium undergoes slight changes in volume or when the intrinsic impedance of cells change as a result of physiological responses.

In addition, as with most customized setups used currently, the stimulation chambers have a large profile, and may occupy a large volume inside the cell culture incubators. Conducting long-term higher throughput studies require a number of discrete components with many wires running into and out of the incubators. In order to circumvent these challenges, a miniature stimulation device with on-board integrated circuits (ICs) and a biocompatible cell array was proposed. Such a device should also allow the output of non-identical waveforms into each cell chamber through the functions of on-board processing ICs.

### Frequency divider circuit design and simulations

**Fig. 5:**
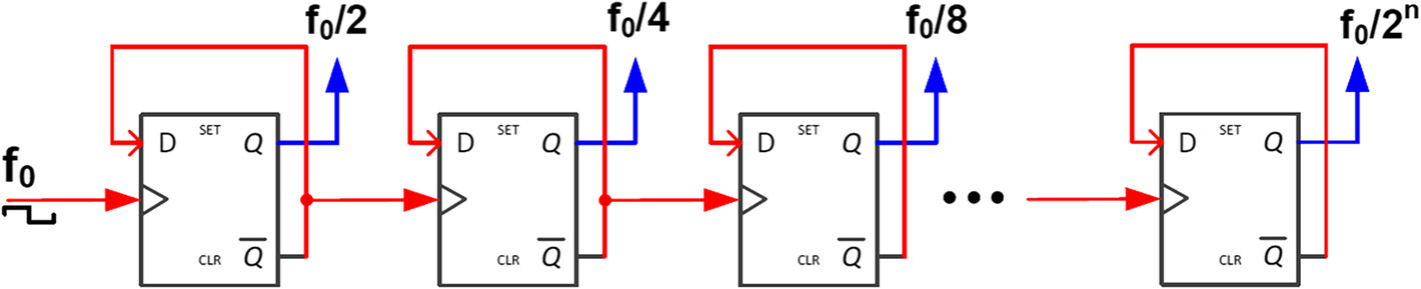
Block diagram of the frequency divider design. Each D-flip-flop is used to realize a “divide-by-2” circuit by connecting the output $\overline{Q}$ to its own input D. For example, clock input with a frequency of f_0_ is fed into the first flip-flops to generate f_0_/2. This f_0_/2 is again used to clock the second flip flop and generate f_0_/4. The sequence can be extended indefinitely. If implemented on-chip, both circuit area and power consumption will be very small and thus can be powered by a miniature battery.

**Fig. 6:**
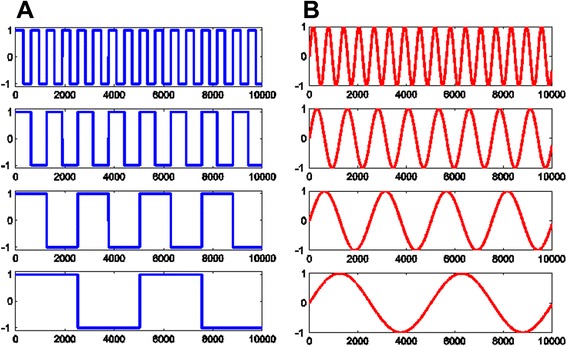
The frequency division of the CMOS 65nm technology simulated using Cadence®. **a** Input frequency of AC square waves (top) is divided by two (second row) then by four (third row), etc. All output waveforms are square waves. **b** If sine waves are desired, these output signals can be passed through a RC low-pass filter (not shown). Sine waveforms with the same frequency can then be obtained as shown.

A frequency divider IC based on the 65 nm complementary metal oxide semiconductor (CMOS) technology was designed by implementing a series of digital flip-flops (Fig. 5). The 65 nm process was selected for its low power consumption and small circuit area, which is suitable for a miniaturized stimulation device when implemented. In such a configuration, the the Q output of each flip-flop is fed into the input of the next flip-flop at half the frequency of the input pulses f_0_. The circuit is designed using the Cadence® design environment and simulations were carried out to test its working principle (Fig. 6). The frequency of input square waveforms was divided by 2^*n*^ in subsequent *n* flip-flops, where each flip-flop will provide the output of f_0_/2^*n*^ to realize a “divide-by-2” circuit (Fig. 6a). As sinusoidal stimulation waveforms are also the target of investigation, they can be obtained at the same frequency by using RC low-pass filters at the end of each flip-flop output (Fig. 6b).

### Cell chamber array fabrication and integration into miniature stimulation device

**Fig. 7:**
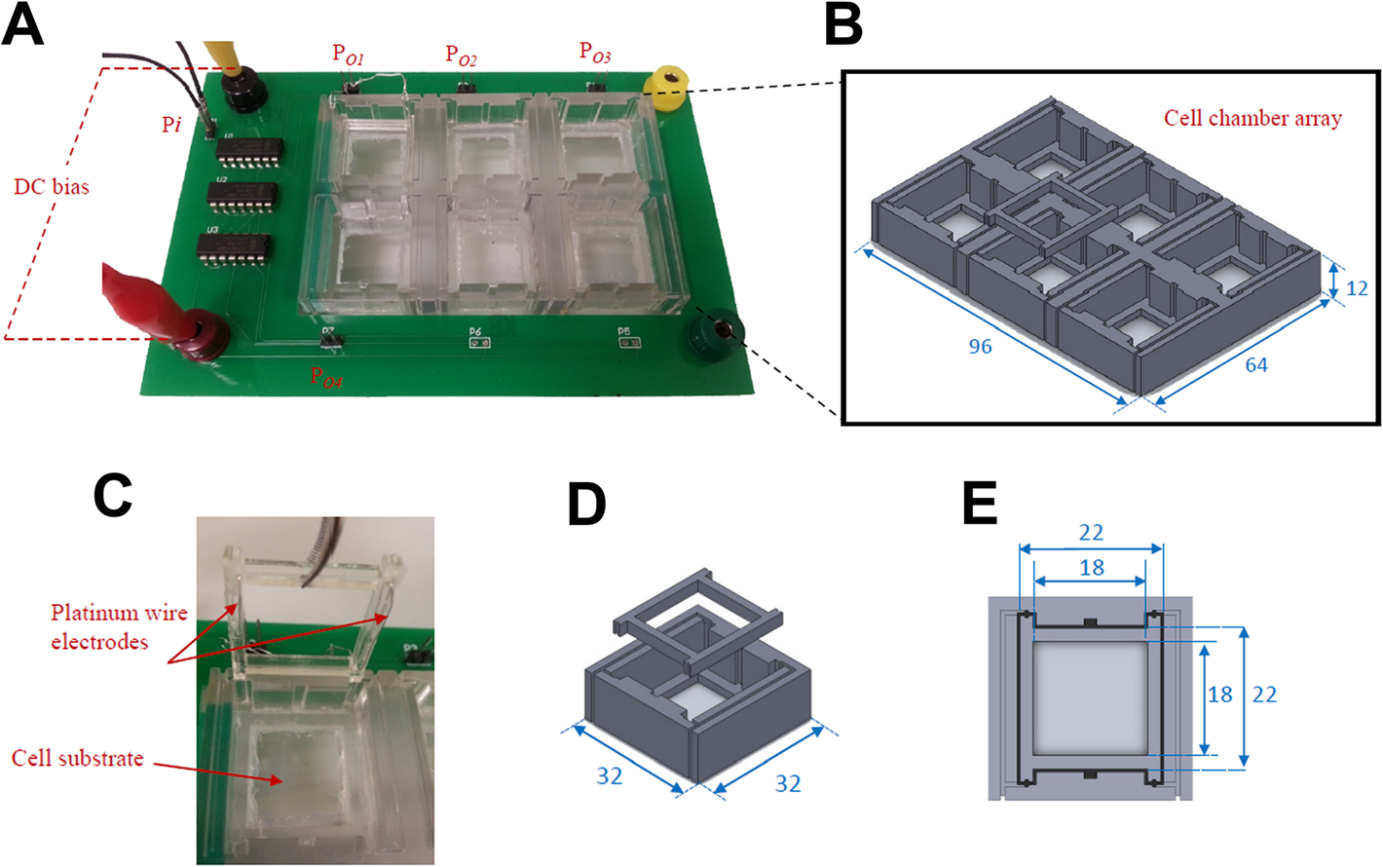
Design of the miniature stimulation device. **a** Whole device with DC bias connectors, waveform input pin P_*i*_ , output pins P_*O1*_-P_*O4*_ and biocompatible cell chamber array, **b** Schematic of integrated cell chamber array, **c** Wire holder lifted out of single cell chamber with a pair of secured platinum wire electrodes, **d** Schematic of single of cell chamber and wire holder, and **e** Top-down schematic of fitted wire holder. All dimensions are in mm.

The 6-well array chamber was designed using CAD software (SolidWorks, USA) and outsourced to a commercial service provider (Prototype Asia, Singapore) for printing using an Objet Polyjet 3D printer (Stratasys Ltd, USA). The biocompatible polymer used was a proprietary UV-curable poly(methyl methacrylate) (PMMA)-based material (VeroClear). The cell chamber was printed in two separate components for assembly: 1) a base frame making up the walls of the 6-chamber array (Fig. 7b), and 2) 6 individual wire holders with notches at 4 corners (Fig. 7c–e). A glass coverslip was attached to the bottom of each chamber of the base frame with Silastic® medical adhesive silicone (Dow-Corning, USA) to create a transparent window that allows for fluorescence microscopic observation of the cells under stimulation. A pair of platinum wires was threaded around the notches and under each wire holder piece to function as a pair of electrodes. A cell substrate measuring 22 × 22 mm in diameter (e.g. another glass coverslip or a conductive material cultured with cells), could be placed in each chamber (Fig. 7c). The wire holder with electrodes was then pushed into the chamber to be held in place. When a conductive or redox-active substrate is used, the wire holder ensures a tight contact between the electrodes and underlying substrate.

### Miniature stimulation device performance

**Fig. 8:**
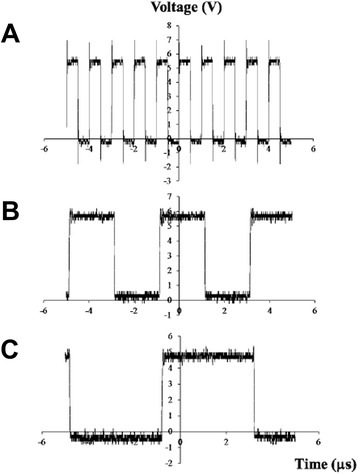
Demonstration of the frequency dividing function of stimulation device. **a** 1 MHz input signal (f_0_) at pin P_*i*_, and recorded output signals at pins **b** P_*O3*_ at 250 kHz (f_0_/4) and **c** P_*O4*_ at 125 kHz (f_0_/8).

The stimulation device was connected to a waveform function generator (AFG3022 Dual Channel, Tektronix Inc, USA) via input pin *P*_*I*_ on the stimulation circuit board to supply 5 V, 1 MHz square waves to the platinum wire electrodes in each chamber (Fig. 8). A 3.3 V DC supply voltage was provided by a DC power supply. An oscilloscope (DSO1022A, Dual Channel, Agilent Technologies, USA) was connected in parallel to voltage output pins *P*_*O1*_, *P*_*O2*_ and *P*_*O3*_ to record the processed output signals. Measured outputs from the platinum electrodes in the cell chamber using an oscilloscope demonstrated the f_0_/2^n^ division function using 5 V square waves. Using the input of 1 MHz square voltages, the output waveforms at *P*_*O3*_ (f_0_/4) and *P*_*O4*_ (f_0_/8) were clean square waves that closely match the simulation results (Fig. 6).

### DC frequency response of ITO-coated glass used with stimulation device

As various studies, including ours, have shown that cellular behaviour can be regulated by material surface chemistry [35–38], one area of interest will be to study the influence of chemical functional groups on materials and if any demonstrated effects are in synergism with electrical stimuli. Some cell stimulation studies have been conducted by applying electrical potentials on cells seeded directly on redox-active materials such as polypyrrole [25] and indium tin oxide (ITO) [39] to investigate the effects of surface charge on biological behaviour e.g. cell adhesion and cell spreading. In light of these studies, one criteria that was incorporated into the design of the stimulation device was to enable users to use a conductive or electroactive substrate seeded with cells as the working electrode. Fitting the platinum wire under the wire holder and pushing the wire holder down into the cell chamber ensured that the platinum wire could be interfaced tightly with substrate (Fig. 7c).

**Fig. 9:**
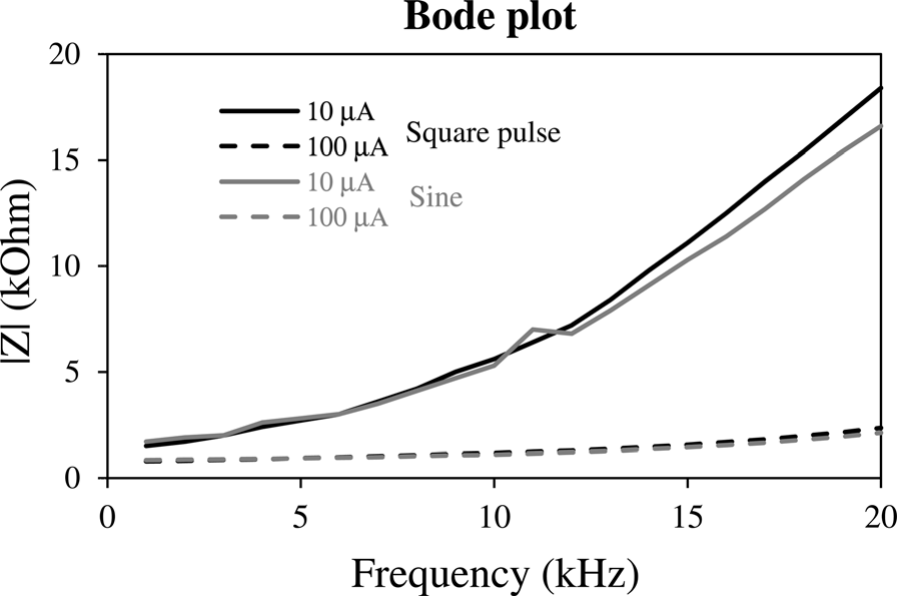
Impedance frequency response Bode plot of ITO-coated glass coverslip. The impedance frequency response was measured with 10 μA and 100 μA square pulse and sine waveforms.

The stimulation device can be used with another conductive substrate to provide material-mediated stimulation for the investigation of biological responses. In this case, a transparent indium tin oxide (ITO)-coated glass (22 mm × 22 mm) was placed in the cell chamber before the wire holder with platinum electrode is pushed down into the chamber. A tight contact was formed between the platinum wire electrode and the gold-plated contacts along the width of the ITO-coated glass. The cell chamber was filled with 1 mL of culture medium to simulate cell culture conditions. The DC power supply was connected to provide the DC power, and the waveform function generator was used to output square pulsed and sine waveforms. A multimeter was connected in series with the setup to record the root-mean-square (r.m.s.) stimulation current. To obtain the frequency response of the ITO-coated glass in cell culture medium, the frequency of the input AC signals was adjusted from 1–20 kHz, and at each frequency point, the amplitude of the peak-to-peak voltage that was needed to keep the r.m.s. current at either 10 μA or 100 μA was recorded. The stimulation r.m.s. currents of 10 μA and 100 μA were chosen as current-mode stimulation at such intensities have demonstrated novel physiological outcomes i.e. mesenchymal stem cell differentiation. The frequency response Bode plots of the system were plotted as shown in Fig. 9 for square pulse and sine waveforms from 1–20 kHz. It can be observed the behaviour of an ITO-coated substrate-cell culture medium system is such that its total impedance modulus increases with high frequency. This behaviour has implications for circuit design to use high frequencies for voltage- or current-controlled mode stimulation with conductive substrates in culture medium.

## Conclusions

The aim of this study was to demonstrate the use of various modes of electrical stimulation in guiding cellular behaviour, and subsequently develop a miniaturized cell stimulation platform to enable research on physiologically-relevant stimulation signals. In parallel with what is known in the literature, cell calcium signaling, polarization of Akt, and cell elongation were demonstrated with discrete components. The stimulation device for performing frequency division of input AC signals to a cell array was designed to enable higher throughput where multiple stimulation frequencies can be applied simultaneously in the same study. In order to perform substrate-based stimulation, the electronic properties of the substrate have to be understood. In this study, the impedance frequency response of conductive ITO-coated glass was characterized using the stimulation device. The miniature stimulation device can be implemented for further cellular studies on cell-material interaction and synergism with electrical stimulation. This modular design consisting of the circuit board layout and 3D-printed cell chambers has the potential to address systematically the effects of different stimulation parameters on cell behaviour. More complex signal-processing ICs can also be implemented on this platform in future.

## Methods

### Materials

SH-SY5Y neuroblast cells (ATCC® CRL-2266™) were purchased from ATCC (Manassas, VA), while human umbilical vein endothelial cells (HUVECs) and EndoGRO-LS complete medium were purchased from Merck Millipore (Billerica, MA). Dulbecco’s Modified Eagle’s Medium (DMEM) was obtained from Sigma-Aldrich (St. Louis, MO). Fetal bovine serum (FBS) and X-Rhod-1 AM calcium-binding dye were ordered from Life Technologies (Carlsbad, CA). FuGene®6 transfection reagent was purchased from Promega Corporation (Madison, WI).

### Pulsed voltage stimulation of SH-SY5Y cells

**Fig. 10:**
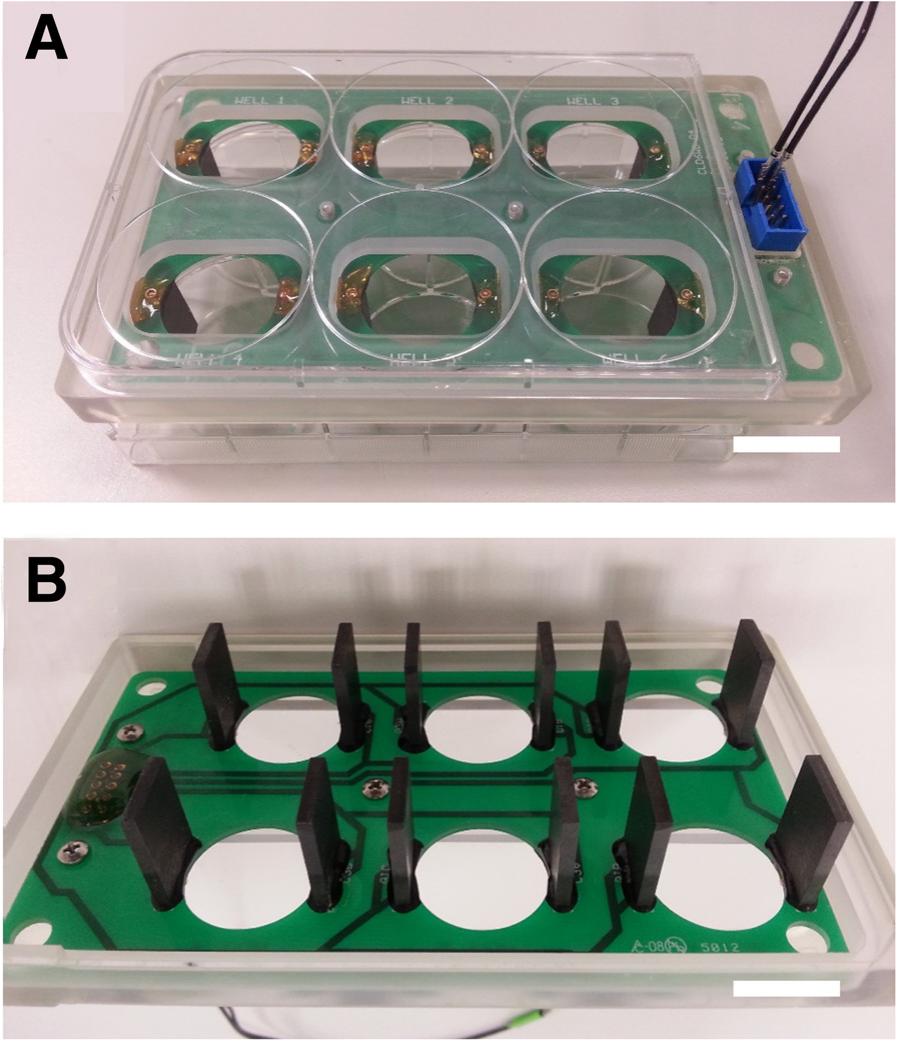
C-Dish™ electrode board adapted for use in cell stimulation. **a** Top view of board on 6-well plate and pin connectors to external waveform generator for single chamber stimulation, and **b** Bottom view of board with carbon electrode pair for each cell chamber and underlying circuit pathways. Scale bar = 20 μm

SH-SY5Y neuroblast cells were stimulated using a commercially-available circuit board with electrodes that allow stimulation experiments to be performed in standard 6-well cell culture dishes (Fig. 10). The SH-SY5Y cells were cultured in DMEM with 10 % FBS. 8.0 × 10^5^ cells were seeded in a 6-well plate and electrical stimulation was performed with the C-dish™ carbon electrode board from IonOptix (Milton, MA). The electrodes of the C-dish™ unit were connected to an external waveform generator through pin connectors. Before stimulation, the cells were loaded with a final concentration of 2 μM of X-Rhod-1 AM calcium-binding dye in calcium-free Tyrode’s solution for 30 min. After removal of staining solution and washing, the cell chamber was filled with 1 mL for Tyrode’s solution supplemented with 2 mM Ca^2+^ ions. The cells were then stimulated with a 5 V monophasic waveform of 1 Hz, 20 msec square pulses under a fluorescence microscope (Axio Observer.Z1, Carl Zeiss, Germany). Images were taken with the Cy 3 filter set (Ex: 640/30, Em: 690/50) before the stimulation, and at 15 min and 30 min after onset of stimulation. The stimulation was then discontinued, and images were taken 15 min post-stimulation.

### DC stimulation of endothelial cells

HUVECs were cultured in EndoGRO-LS complete medium. 1.0 × 10^6^ cells were seeded in each well of the 6-well plate. A DC power supply was connected to the C-Dish™ electrode board to generate the required DC current. A 100-Ohm resistor was connected in series with the waveform generator to determine the DC current though 1 mL of cell culture medium (calculated from the voltage drop across the resistor and Ohm’s law, R = V/I). A current of 1.5 mA was set for the stimulation through tuning of the DC voltage. The stimulation was carried out for 12 h in a CO_2_ incubator at 37 °C. The morphology of the HUVECs were observed under a light microscope (AxioVert, Carl Zeiss, Germany) and phase contrast images were taken using the camera attachment. The elongation of HUVEC cells was quantitated by measuring the long and short axis of 10 cells from each field before stimulation and after 12 h stimulation, and expressed as a ratio of long/short axis.

### DC stimulation of *PH-Akt-GFP* transfected keratinocytes

The human keratinocyte cell line HaCaT was cultured in DMEM with 10 % FBS and maintained at less than 50 % confluency. 5 × 10^4^ cells were then seeded on a glass coverslip and allowed to reach 50 % confluency. A pCMV vector with the *PH-Akt-green fluorescence protein (GFP)* construct was transfected into HaCaT cells using FuGene®6 transfection reagent following the manufacturer’s protocol. 24 h post-transfection, the glass coverslips were transferred to a 6-well plate and cells were checked for green fluorescence protein (GFP) expression under the fluorescence microscope. The C-dish™ electrode board connected to a DC power supply was used to provide a constant voltage of 150 mV/mm across each well. A single cell expressing *PH-Akt* was focused on and images were taken using the GFP filter set (Ex: 470/40, Em: 525/50) every 15 min for 1 h to monitor the redistribution of *PH-Akt-GFP* within the cell. The total signal intensity of each cell, as well as a region of interest (ROI) was marked around the anodal end of the cell, and measured in ImageJ. The localization of *PH-Akt-GFP* at the anodal was then determined from the proportion of signal intensity in the ROI/the total signal intensity in the whole cell.
